# Concerns Regarding a New Culture Method for *Borrelia burgdorferi* Not Approved for the Diagnosis of Lyme Disease

**Published:** 2014-04-18

**Authors:** Christina Nelson, Sally Hojvat, Barbara Johnson, Jeannine Petersen, Marty Schriefer, C. Ben Beard, Lyle Petersen, Paul Mead

**Affiliations:** 1 Division of Vector-Borne Diseases, National Center for Emerging and Zoonotic Infectious Diseases, CDC; 2 Division of Microbiology Devices, Office of In Vitro Diagnostics and Radiological Health, Center for Devices and Radiological Health, FDA

In 2005, CDC and the Food and Drug Administration (FDA) issued a warning regarding the use of Lyme disease tests whose accuracy and clinical usefulness have not been adequately established (1). Often these are laboratory-developed tests (also known as “home brew” tests) that are manufactured and used within a single laboratory and have not been cleared or approved by FDA. Recently, CDC has received inquiries regarding a laboratory-developed test that uses a novel culture method to identify *Borrelia burgdorferi*, the spirochete that causes Lyme disease. Patient specimens reportedly are incubated using a two-step pre-enrichment process, followed by immunostaining with or without polymerase chain reaction (PCR) analysis. Specimens that test positive by immunostaining or PCR are deemed “culture positive” (2). Published methods and results for this laboratory-developed test have been reviewed by CDC. The review raised serious concerns about false-positive results caused by laboratory contamination and the potential for misdiagnosis (3).

CDC recommends that laboratory tests cleared or approved by FDA be used to aid in the routine diagnosis of Lyme disease. A complete searchable list of such tests is available online (4).

When evaluating testing options, providers and their patients might be confused by the distinction between Clinical Laboratory Improvement Amendments (CLIA) certification of laboratories and FDA clearance or approval of specific tests. CLIA certification of a laboratory indicates that the laboratory meets a set of basic quality standards.* It is important to note, however, that the CLIA program does not address the clinical validity of a specific test (i.e., the accuracy with which the test identifies, measures, or predicts the presence or absence of a clinical condition in a patient).† FDA clearance/approval of a test, on the other hand, provides assurance that the test itself has adequate analytical and clinical validation and is safe and effective.§

When laboratory testing is indicated, CDC recommends two-tier serologic testing for the diagnosis of Lyme disease. Two-tier testing consists of an FDA-cleared enzyme immunoassay (EIA) that, if positive or equivocal, is followed by an FDA-cleared immunoblot test, commonly known as a “Western blot” test. Results are considered positive only when both the EIA and Western blot are positive (5). Culture and PCR of clinical specimens are recommended only in certain rare circumstances (6).

CDC encourages researchers to work with FDA to develop new or improved tests for the diagnosis of Lyme disease. As with any diagnostic test, it is critical that new tests for Lyme disease have adequate analytical and clinical validation to avoid misdiagnosis and improper treatment of patients.

## Footnotes

* 42 U.S.C. §263a; 42 CFR Part 493.

† Additional information available at http://www.cms.gov/regulations-and-guidance/legislation/clia/downloads/ldt-and-clia_faqs.pdf.

§ 21 U.S.C. §§360c, 360e and 21 CFR814.20, 860.7.
